# Comparison of clinical outcomes, demographic, and laboratory characteristics of hospitalized COVID‐19 patients during major three waves driven by Alpha, Delta, and Omicron variants in Tehran, Iran

**DOI:** 10.1111/irv.13184

**Published:** 2023-08-09

**Authors:** Zahra heydarifard, Nazanin‐Zahra Shafiei‐Jandaghi, Moslem Safaei, Forough Tavakoli, Somayeh Shatizadeh Malekshahi

**Affiliations:** ^1^ Hepatitis Research Center, Department of Virology, Faculty of Medicine Lorestan University of Medical Sciences Khorramabad Iran; ^2^ Virology Department, School of Public Health Tehran University of Medical Sciences Tehran Iran; ^3^ Department of Pharmacy, School of Pharmacy Shahid Sadoughi University of Medical Science Yazd Iran; ^4^ Department of Bacteriology and Virology, School of Medicine Isfahan University of Medical Sciences Isfahan Iran; ^5^ Department of Virology, Faculty of Medical Sciences Tarbiat Modares University Tehran Iran

**Keywords:** COVID‐19, epidemiology, Iran, mortality, variants, waves

## Abstract

**Introduction:**

This study is the first study in which demographic, laboratory data, and outcomes of coronavirus disease‐2019 (COVID‐19) patients due to the circulating SARS‐CoV‐2 infections caused by different variants (Alpha, Delta, and Omicron) are compared in Iran.

**Methods:**

We conducted a retrospective study of confirmed hospitalized COVID‐19 cases from April 9, 2021, to May 22, 2022. Demographic data and laboratory findings were extracted from patients' electronic medical records on the first day of admission to the hospital. All patients were followed up for outcomes related to COVID‐19 including intensive care unit (ICU) admission and mortality rate.

**Results:**

Of 760 confirmed hospitalized COVID‐19 cases, 362, 298, and 100 represented patients during waves 4–6, respectively. During the Omicron wave, hospitalized patients were older than the other two waves and had a lower median level of C‐reactive protein (CRP), alanine transaminase (ALT), aspartate transaminase (AST), and erythrocyte sedimentation rate (ESR). The median length of hospital stay during waves 4–6 was 5 days (interquartile range [IQR]: 4.0–8.0), 7 days (IQR: 6.0–11), and 6 days (IQR: 5.0–9.0), respectively (*p* < 0.001). The rate of ICU admission during waves 4–6 significantly increased.

**Conclusions:**

Although the Omicron variant caused less severe disease, in older patients who were hospitalized due to Omicron infection, longer hospital and ICU stays were reported, which could be attributed to their old age. In particular, elderly patients are more vulnerable to severe COVID‐19; otherwise, as expected, other laboratory parameters and clinical outcomes were in accordance with differences in pathogenicity and infectivity of these variants.

**Abbreviations**
ACE2: angiotensin‐converting enzyme 2
ALT: alanine transaminase
AST: aspartate transaminase
COVID‐19: coronavirus disease‐2019
CRP: C‐reactive protein
ESR: erythrocyte sedimentation rate
IQR: interquartile range
RBC: red blood cell
RBD: receptor binding domain
SARS‐CoV‐2: severe acute respiratory syndrome coronavirus 2
VOCs: variants of concern
WBC: white blood cell count

## 1 BACKGROUND

The world has faced a major public health challenge due to the severe acute respiratory syndrome coronavirus 2 (SARS‐CoV‐2) as the cause of coronavirus disease‐2019 (COVID‐19) and still, it is unclear when the pandemic will come to a complete end.^1^ Until August 8, 2022, Iran has experienced seven waves of COVID‐19 after the first confirmed case of SARS‐CoV‐2 in Iran on February 19, 2020.^2^ Genetic analyses have revealed that the SARS‐CoV‐2 genome appears to be evolving relatively more slowly than others such as influenza viruses or HIV.^3^ Gradually, several variants of SARS‐CoV‐2 have been introduced showing genetic differences from the original Wuhan strain, some of which are described as variants of concern (VOCs).^4^, ^5^ VOCs are attributed to induce multiple adverse consequences such as an increase in transmissibility or virulence, reduction in naturalizing antibodies, the ability to evade detection, and reduced effectiveness of therapeutics or vaccines. Alpha, Beta, Gamma, Delta, and Omicron are classified as VOCs since the beginning of the pandemic.^4^ Previous studies in Iran compared the first three waves of COVID‐19 according to severity, intensive care unit (ICU) admission, and mortality rate with the main focus on the Alpha and Beta variants.^6^, ^7^ Nevertheless, to the best of our knowledge, no study describes the patients' characteristics in recent waves in the country driven by different variants. The fourth wave in Iran began in early April–June 2021, and the fifth wave continued from August to October 2021. The sixth wave was from late January 2022 until now.^8^ The dominant variant circulating during the fourth, fifth, and sixth waves of the COVID‐19 in Iran was Alpha, Delta, and Omicron, respectively.^8^ These waves of COVID‐19 impose a more significant hospitalization and mortality rate than earlier waves. The fifth wave in August 2021 was one of the country's most devastating episodes of the pandemic, according to the report.^9^ One observational study in Spain showed that patients' characteristics varied between different waves.^10^ Currently, Omicron is the predominant variant circulating throughout the world. However, the severity and in‐hospital outcomes of this variant are not well characterized.^11^ Numerous studies have explored the risk factors of SARS‐CoV‐2 infection and reported severity and long‐term hospitalization associated with laboratory parameters such as elevated levels of creatinine, urea, and C‐reactive protein (CRP).^12^, ^13^

This aim of this study is to describe the demographic data, laboratory parameters, and different in‐hospital outcomes across the last three major waves of COVID‐19 driven by Alpha, Delta, and Omicron variants in hospitalized patients in Tehran, Iran.

## 2 METHODS

### 2.1 Study design and participants

We performed a single‐center retrospective study of proven hospitalized COVID‐19 cases in Valiasr Hospital, a COVID‐19 referral center affiliated with Shahid Beheshti University of Medical Sciences (SBUMS) in Tehran, Iran, from April 9, 2021, to May 22, 2022.

The inclusion criteria for this study were (I) patients over 20 years of age diagnosed with COVID‐19 by real‐time polymerase chain reaction (PCR) and (II) those admitted to the hospital according to moderate to severe COVID‐19 infection. We defined moderate and severe patients according to standard guidelines. Moderate patients are considered to have lower respiratory tract involvement with oxygen saturation (SpO_2_) ≥ 94% on room air at sea level. Patients with oxygen saturation (SpO_2_ < 94%) on room air, oxygen partial pressure (PaO_2_)/fractional inspired oxygen (FiO_2_) ratio < 300 mmHg, a respiratory rate > 30 breaths/min, or lung infiltrates > 50% are defined as severe COVID‐19 infection. We also included other criteria such as the need for intubation and admission to the ICU, which were monitored throughout the patients' hospitalization. Patients who were not admitted for COVID‐19 and children younger than 20 were excluded from the study. All patients in three waves received glucocorticoid and anticoagulant therapy.

Demographic data and laboratory findings were extracted from patients' electronic medical records on the first day of admission to the hospital. All patients were followed up for outcomes related to COVID‐19 including ICU admission and mortality rate. We categorized patients into the last three waves based on reports that recognized distinct waves in different months in Iran.^8^, ^14^ The fourth wave in Iran was defined as the period from April 9 to June 10, 2021, the fifth wave was defined as the period between August 9 and October 10, 2021, and the sixth wave was defined as the period between January 20 and May 22, 2022. Variant‐specific PCR was used for the validation of SARS‐CoV‐2 variants in the community.

### 2.2 Statistical analysis

Categorical variables were presented as counts and frequencies and then compared using the chi‐square test. To assess the normal distribution of numerical variables, we used the Kolmogorov–Smirnov and Shapiro–Wilk tests. Normally distributed numerical variables were presented as mean ± SD and compared using the one‐way analysis of variance (ANOVA) test. Variables with skewed distributions were presented as median (interquartile range [IQR]) and compared using the Kruskal–Wallis test. All statistical analyses were conducted using SPSS Statistics for Windows Version 21. We considered *p* < 0.05 to be statistically significant.

## 3 RESULTS

### 3.1 Demographic characteristics

Seven hundred sixty patients hospitalized with COVID‐19 infection were enrolled in the study. Of 760 included patients, 362, 298, and 100 represented patients during waves 4–6, respectively. A total of 102 (13.4%) patients were deceased during this study. Moreover, there were 113 (14.9%) admissions to the ICU and 647 (85.1%) to the wards. The majority of those who died (49 out of 102 [48%]) were over the age of 70. During the fourth, fifth, and sixth waves (in which Alpha, Delta, and Omicron were as the dominant circulating variant, respectively), the mean ages of hospitalized patients were 58 ± 15.7, 55.5 ± 15.6, and 67.4 ± 17.5, respectively (*p* < 0.001). Men accounted for the majority of COVID‐19 cases (411 out of 760 [54.2%]) during the study period. However, patients did not differ based on sex among the three waves (*p* = 0.928).

### 3.2 Clinical characteristics and laboratory data

Clinical presentation among hospitalized patients with COVID‐19 did not show significant differences between these three waves. However, the most frequently reported clinical symptoms were fever, dyspnea, cough, chest pain, myalgia, and headache (not shown). The laboratory findings at admission are outlined in Table 1. There were significant differences in CRP (*p* < 0.001), blood urea (*p* = 0.001), creatinine (*p* = 0.01), aspartate transaminase (AST) (*p* = 0.008), alanine transaminase (ALT) (*p* < 0.001), white blood cell count (WBC) (*p* = 0.005), red blood cell (RBC) (*p* = 0.001), platelet, neutrophil‐to‐lymphocyte ratio (*p* < 0.001), and magnesium (*p* < 0.001) levels of patients between the waves. However, blood sugar (*p* = 0.3) and calcium (*p* = 0.06) were not significantly different in patients admitted in the three waves. Statistical analysis showed that patients in wave 6 had significantly higher urea, creatinine, neutrophil‐to‐lymphocyte ratio, and platelet and lower CRP, AST, and ALT than the two other waves.

**TABLE 1:** Baseline characteristics and in‐hospital outcomes of hospitalized COVID‐19 patients during different waves.

Characteristic	4th wave	5th wave	6th wave	Total	*p*‐value
**Demographics**					
Age (years)	58.0 ± 15.7	55.5 ± 15.6	67.4 ± 17.5	58.2 ± 16.3	**<0.001**
Sex					
Male	198 (54.8%)	160 (53.7%)	53 (53.0%)	411 (54.2%)	0.9
Female	163 (45.2%)	138 (46.3%)	47 (47.0%)	348 (45.8%)	
**Laboratory data on admission**					
WBC (×$10^{3}$/μL)	5.7 [4.2–8.2]	5.6 [4.3–8.6]	7.1 [5.0–10.1]	5.8 [4.3–8.6]	**0.005**
Neutrophil (×$10^{3}$/μL)	5.2 [3.6–7.9]	5.1 [3.2–7.8]	6 [3.2–8.0]	5.3 [3.2–7.7]	**0.001**
Lymphocyte (×$10^{3}$/μL)	1.1 [0.7–1.2]	1.2 [1.0–1.5]	1.0 [0.6–1.3]	1.2 [0.9–1.5]	**<0.001**
Neutrophil‐to‐lymphocyte ratio	5.2 [4.8–6.7]	4.6 [3.0–5.3]	6.4 [5.1–6.6]	4.3 [3.2–5.2]	**<0.001**
Platelets (×$10^{3}$/μL)	175 [134.0–219.0]	167 [127.0–209.0]	189 [138.0–237.0]	173 [132.0–220.0]	**0.04**
RBC (cell/μL)	4.8 [4.4–5.2]	4.8 [4.4–5.2]	4.5 [4.0–5.0]	4.7 [4.4–5.2]	**0.001**
CRP (mg/L)	49.7 [23.4–71.8]	51.4 [27.1–84.4]	28.1 [11.0–51.0]	48.9 [22.9–72.1]	**<0.001**
BS (mg/dL)	117 [97.0–154.0]	124 [97.0–177.0]	124 [98.0–164.0]	123 [97.0–161.0]	0.3
Urea (mg/dL)	36 [27.0–47.0]	36 [28.0–52.0]	44 [31.0–60.0]	37 [28.0–51.0]	**0.001**
Creatinine (mg/dL)	1.1 [1.0–1.3]	1.1 [1.0–1.3]	1.2 [1.0–1.6]	1.1 [1.0–1.3]	**0.01**
Calcium (mmol/L)	8.6 [8.2–9.0]	8.7 [8.2–9.0]	8.8 [8.4–9.2]	8.7 [8.2–9.0]	0.06
Magnesium (mmol/L)	2.4 [2.1–2.6]	2.1 [1.9–2.3]	2.1 [1.8–2.4]	2.2 [2.0–2.4]	**<0.001**
AST (U/L)	40 [31.0–53.0]	41 [28.0–63.0]	33.5 [25.0–48.0]	40 [29.0–56.0]	**0.008**
ALT (U/L)	28 [19.0–48.0]	36 [23.0–57.0]	26 [16.0–37.0]	32 [20.0–51.0]	**<0.001**
ESR (mm/h)	52 [28.0–71.0]	57 [35.0–86.0]	50 [26.0–70.0]	54 [27.0–90.0]	**<0.001**
**In‐hospital outcomes**					
Hospital length of stay (days)	5 [4.0–8.0]	7 [6.0–11.0]	6 [5.0–10.0]	6 [5.0–9.0]	**<0.001**
ICU admission	47 (13.0%)	43 (14.4%)	23 (23.0%)	113 (14.9%)	**0.04**
ICU length of stay (days)	6 [8.0–9.0]	5 [5.0–7.0]	12 [7.0–16.0]	6 [3.5–11.0]	**0.02**
Mortality	45 (12.4%)	43 (14.4%)	14 (14.0%)	102 (13.4%)	0.7

*Note*: Data are presented as mean ± standard deviation, number (%), or median [interquartile range]. Statistically significant *p*‐values are bolded.

Abbreviations: ALT, alanine transaminase; AST, aspartate transaminase; BS, blood sugar; COVID‐19, coronavirus disease‐2019; CRP, C‐reactive protein; ESR, erythrocyte sedimentation rate; ICU, intensive care unit; RBC, red blood cell; WBC, white blood cell count.

### 3.3 In‐hospital outcomes and clinical complications

**FIGURE 1:**
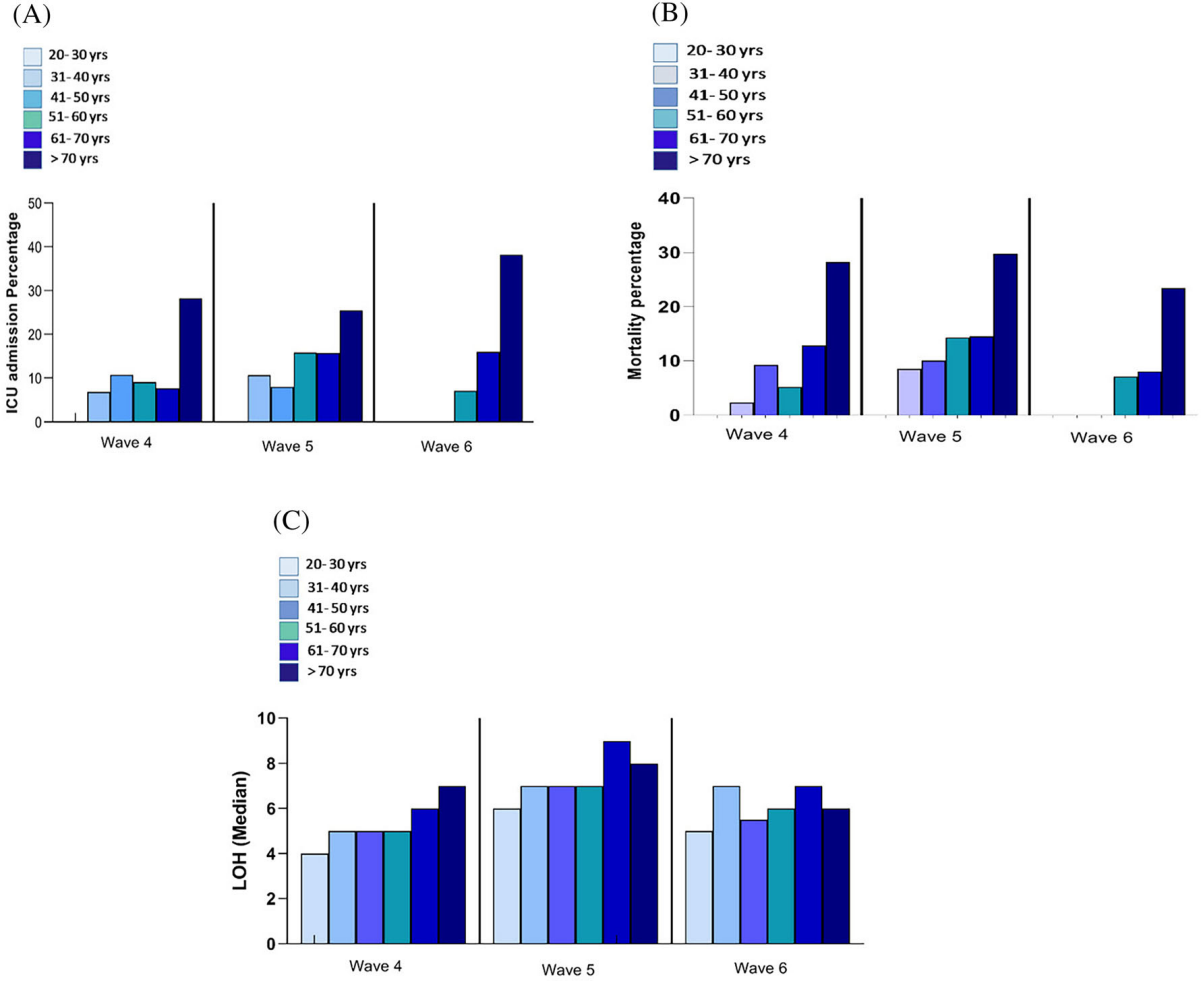
The percentage of intensive care unit (ICU) admission (A), deceased patients (B), and length of hospital stay (LOH) (C) by age group during three waves, one hospital, Tehran, Iran, from April 9, 2021, to May 22, 2022.

The median length of hospital stay for the fourth, fifth, and sixth waves was 5 days (IQR: 4.0–8.0), 7 days (IQR: 6.0–11), and 6 days (IQR: 5.0–9.0), respectively (*p* < 0.001). The hospital stay was longer in wave 5 than in the other two waves. There was no significant difference in mortality rate from waves 4 to 6, with 45 (12.4%), 43 (14.4%), and 14 (14%) deaths, respectively (*p* = 0.7). However, the number of deceased patients during wave 5 was slightly more than in the other two waves. The rate of ICU admission during waves 4–6 significantly increased. Subsequently, the ICU admission rate during waves 4–6 was 13%, 14.4%, and 23%, respectively (*p* = 0.04). Comparing ICU length of stay showed that the ICU length of stay during wave 6 was longer than the other ones (*p* = 0.008). The percentage of ICU, deceased patients, and length of hospital stay for the three waves are presented in Figure 1.

## 4 DISCUSSION

As far as we know, this observational study is the first to compare demographic, laboratory data, and outcomes of COVID‐19 patients due to the current SARS‐CoV‐2 infections caused by different variants (Alpha, Gamma, and Delta). Besides, it is the first study in Iran comparing these variables during the fourth, fifth, and sixth waves of COVID‐19. The most noticeable finding of this study was the difference in age, length of hospital stay, ICU admission, and ICU length of stay in the three different waves of COVID‐19 infection. In contrast, there was no difference in in‐hospital mortality among the three different waves. The mean age of hospitalized patients in our study was 58.2 ± 16.3 years. Our study had a higher mean age during the Omicron wave (67.4 ± 17.5) compared with earlier waves, which is in contrast with Yoon et al. and Bouzid et al. who reported that patients infected with Omicron had a lower mean age compared with the Delta variant (62 vs. 67 years and 54 vs. 62 years), respectively.^15^, ^16^ The reasons for this contradiction could be differences in study populations and including both hospitalized and non‐hospitalized patients, whereas in our study, we excluded non‐admitted patients.

The crucial features of the Delta variant are mutations in the spike protein that confer increased affinity for the angiotensin‐converting enzyme 2 (ACE2) receptors. Among these mutations, S:P681R substitution in the furin cleavage site leads to increase virus spread and infectivity.^17^ In addition, researchers around the world reported that Delta variants are associated with higher infectious virus loads,^18^ raised inflammatory factors like CRP,^19^ severe disease profiles,^20^ and high rate of hospital admission^21^ compared with pre‐Delta variants. In this study, it was observed that hospitalization stays due to infection with the Delta variant were longer than those with the Omicron and Alpha variants. This finding is consistent with previous studies that have reported an association between the Delta variant and longer hospital stays.^16^, ^22^, ^23^

The Omicron variant has triple mutations at the furin cleavage site (P681H, H655Y, and N679K) that help it to replicate more and to increase its transmissibility efficiently. The receptor binding domain (RBD) of the viral spike protein has two mutations (T478K and N501Y) associated with increased viral binding affinity. Most mutations are associated with increased pathogenicity and potential evasion of infection‐blocking antibodies.^24^

The early finding suggested that the Omicron variant had a reduced ability to replicate in the lung parenchyma, which may have contributed to a reduced disease severity compared with previous variants.^25^ However, most of the patients hospitalized for COVID‐19 early in the Omicron wave had reduced respiratory symptoms and abnormal chest imaging, about one third had hypoxemia, and 10% required invasive mechanical ventilation. These results reflect that, despite the changes observed compared with Delta, infection with the Omicron variant still causes severe lower respiratory tract disease.^26^

Omicron has milder symptoms than the Delta variant, but older people are still at risk of serious complications because their immune system is not as strong and antibody levels are usually insufficient to fight such mutant viruses.^24^ Interestingly, we observed a greater length of stay in the ICU and a high rate of ICU admission in the elderly group during the Omicron wave compared with the other two waves, which is contrary to Jassat et al.^27^ and Sievers et al.^28^ who showed that ICU admission during the Omicron wave was lower than earlier waves. In our study, most of the patients (more than 70%) who were admitted to the hospital during the Omicron wave were elderly patients. One recent study concluded that elderly were more susceptible to infection with the Omicron variant, even in fully vaccinated individuals.^24^ Therefore, this age group needed more critical care and referral to ICU.^29^ Among admitted elderly patients during the Omicron variant, 78.3% of patients were referred to ICU. Unfortunately, we had no access to underlying disorder data, so it was not clear how many of these patients had comorbidity, which affected COVID‐19 severity.

During the Delta predominance era, a small percentage of Iranians had been fully vaccinated that might be linked to severe symptoms and in‐hospital mortality. In this regard, consistent with previous studies, elevated CRP, erythrocyte sedimentation rate (ESR), ALT, and AST as laboratory parameters for the severity of COVID‐19 were observed in the Delta variant.^30^, ^31^ One probable cause for higher hepatic transaminases could be attributed to adverse effects from multi‐drug therapy during the fifth wave than the other two waves. However, magnesium was slightly higher during Alpha than in the other two waves due to the anti‐inflammatory and anti‐thrombotic effects of Mg^2+^ that were related to reducing the severity of COVID‐19 symptoms.^32^ An increase in urea and creatinine levels during the Omicron wave could be related to increasing age in hospitalized patients during this period.^33^, ^34^

In the sixth wave by the Omicron variant, regardless of vaccination status, the majority of infected individuals were young asymptomatic outpatients while patients with comorbidities and elderly patients had a high risk for severe COVID‐19 and hospitalization.^35^, ^36^ According to this point, in the present study, elderly patients comprised most patients when Omicron was predominant; consequently, the rate of mortality in hospitalized patients has not dramatically declined in comparison with the COVID‐19 cases due to infection with the Delta variant. In accordance with our results, the study was conducted in the United Kingdom and reported a significant increase in hospitalizations and deaths among those ≥75 years during the Omicron variant surge.^37^

The previous studies conducted in Iran have investigated the earlier waves of COVID‐19. A retrospective study by Hadadi et al., which was conducted from February 16 to October 28, 2020, during the first, second, and third waves of COVID‐19, found that the length of hospital stay and acute cardiac diseases increased in these waves. Moreover, Jalali et al. revealed differences in epidemiologic features between the second wave and the first wave of COVID‐19 in Babol, North of Iran.^6^ It is also important to note that the current circulation variant (Omicron variant) was not included in these studies.

It should be mentioned that this study had some limitations that must be emphasized. First, this study was an observational study and has inherent potential bias. Second, some patients' demographic data such as comorbidities were not available so we could not compare these factors between waves. Third, it was a single‐center study on the Iranian population, and future multicenter studies with larger sample sizes are needed.

## 5 CONCLUSIONS

Despite these limitations, herein, observed differences in patients' characteristics (precisely around 10 years of age difference), laboratory tests, and in‐hospital outcomes of admitted patients during three waves of COVID‐19 driven by different variants were in accordance with the characteristics of the variants that caused these COVID‐19 waves. It should be highlighted that the present study was performed on hospitalized COVID‐19 patients. Consequently, even though Omicron variant caused less severe disease, in older patients who were hospitalized due to Omicron infection, longer hospital and ICU stays were reported, which could be attributed to their old age. Also, variation/rise/decline in some of the lab test results and clinical outcomes could be attributed to aging in hospitalized patients with Omicron infection. In particular, elderly patients are more vulnerable to severe COVID‐19; otherwise, as expected, other laboratory parameters and clinical outcomes were in accordance with differences in pathogenicity and infectivity of these variants.

## AUTHOR CONTRIBUTIONS

**Zahra heydarifard:** Investigation; methodology; writing—original draft. **Nazanin‐Zahra Shafiei‐Jandaghi:** Investigation; methodology; review and editing. **Moslem Safaei:** Investigation. **Forough Tavakoli:** Data curation; methodology. **Somayeh Shatizadeh Malekshahi:** Conceptualization; investigation; methodology; writing—review and editing.

## CONFLICT OF INTEREST STATEMENT

The authors declare that there are no conflicts of interest.

### PEER REVIEW

The peer review history for this article is available at https://www.webofscience.com/api/gateway/wos/peer-review/10.1111/irv.13184.

## ETHICS STATEMENT AND CONSENT TO PARTICIPATE

The study was conducted in accordance with the Declaration of Helsinki and national and institutional standards and it was approved by the Valiasr Naja Hospital Medical Ethics Committee. Patients signed written informed consents and accepted the publication of clinical data for research. All methods were performed in accordance with the relevant guidelines and regulations.

## Data Availability

All data are available from the corresponding author upon reasonable request.
